# A rare anomaly of A4+5 on three-dimension multidetector computed tomography in lung cancer: A case report

**DOI:** 10.1016/j.ijscr.2019.08.013

**Published:** 2019-08-20

**Authors:** Shinichi Sakamoto, Hiromasa Matsumoto, Hiroyuki Hino, Shoji Sakiyama

**Affiliations:** Department of Thoracic Surgery, National Hospital Organization Kochi National Hospital, Kochi, Japan

**Keywords:** VATS, video-assisted thoracic surgery, 3D-MDCT, three-dimensional multidetector computed tomography, PET, positron emission tomography, VC, vital capacity vital capacity, FEV1, forced expiratory volume in 1S, PA, pulmonary artery, Video-assisted thoracic surgery, Three-dimension multidetector computed tomography, Anomaly

## Abstract

**Introduction:**

There are several anomalies of the pulmonary vessels. Clinicians need to be well informed about anatomy, particularly before video-assisted thoracic surgery, to prevent fatal complications.

**Presentation of case:**

We report the case of an 80-year-old woman who was suspected of having lung cancer in the right lower lobe. The patient was accordingly scheduled for surgery. Three-dimension multidetector computed tomography (3D-MDCT) showed an extremely rare anomaly in which A4 + 5 ran between V2 and V1 + 3. We scheduled a non-anatomical wedge resection of the lesion and performed rapid pathological diagnosis during surgery. Because adenocarcinoma was diagnosed, we performed right lower lobectomy using video-assisted thoracic surgery. Station 11i lymph node rigidly adhered to the main pulmonary artery, V2, and intermedius bronchus. Thus, the surgery was shifted to middle and lower lobectomy.

**Discussion:**

To the best of our knowledge, this type of anomaly has not been reported yet. Pulmonary vessels can be accurately identified using 3D-MDCT; thus, a rare anatomy can be identified, and information can be shared across the surgical team simulating familiarity with this rare anatomy.

**Conclusion:**

Using 3D-MDCT, we could accurately divide A4 + 5 and safely perform the surgery.

## Highlights

• We report an extremely rare anomaly in which A4 + 5 ran between V2 and V1 + 3.
• Correct anatomy of the pulmonary vessels can be known using preoperative 3D-MDCT.
• Surgery can be smoothly modified using preoperative 3D-MDCT.

## 1 Introduction

In lung surgery, there are several variations in the pulmonary vessels. Anomalies of the pulmonary vessels can cause serious complications, such as bleeding. It is important to understand the correct anatomy to safely perform surgery, especially in case of video-assisted thoracic surgery (VATS). Using three-dimensional multidetector computed tomography (3D-MDCT), the presence of anomalies can be easily confirmed [1]. Herein, we report the case of a patient with an extremely rare anatomy, as observed on 3D-MDCT, who underwent a successful surgery. The work has been reported in line with the SCARE 2018 statement [2].

## 2 Presentation of case

**Fig. 1:**
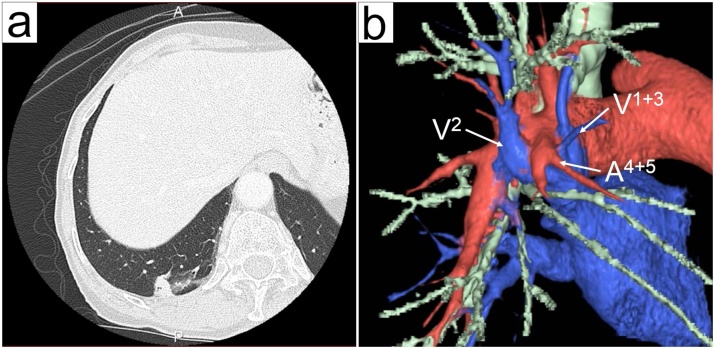
(a) Computed tomography (CT) shows a 15-mm tumor in the right lower lobe. (b) Three-dimensional multidetector CT shows that A4 + 5 is running between V2 and V1 + 3.

In an 80-year-old woman, an abnormal shadow was detected in the right lung field during a routine physical examination. She had no past medical and surgical history. Chest computed tomography (CT) revealed a 15-mm tumor in the right lower lobe that slowly increased in size (Fig. 1). Laboratory examination showed an increase in carcinoembryonic antigen level at 9.2 ng/mL. Bronchoscopy was performed; however, no signs of malignancy and abnormalities in the branching pattern of the bronchial trees were observed. Further, 18F-fluorodeoxyglocose positron emission tomography (PET) revealed an abnormal uptake only in station 11i lymph node (standardized uptake value: 5.1), whereas no marked accumulation was observed in the right lower mass. Pulmonary function tests showed that vital capacity (VC) was 2090 ml, the percentage of predicted VC was 100.0%, forced expiratory volume in 1S (FEV1) was 1510 ml, and FEV percentage in 1S was 104.1%. Therefore, we scheduled wedge resection using VATS for surgical diagnosis and treatment. We planned to perform the surgery using 3D-MDCT based on MDCT using the Fujifilm Synapse Vincent system (Fujifilm Corporation, Tokyo, Japan). 3D-MDCT images revealed an anomaly wherein A4 + 5 ran between V2 and V1 + 3.

**Fig. 2:**
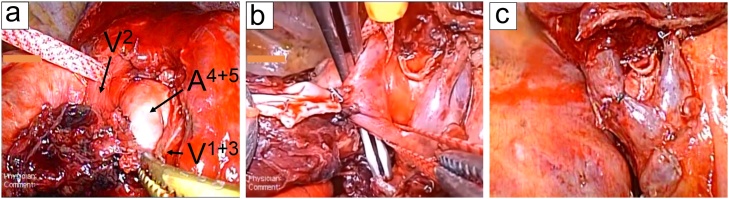
(a) Station 11i lymph node (#11i) has rigidly adhered to the intermedius bronchus, V2, and common basal PA. A4 + 5 is running between V2 and V1 + 3. (b) We performed V2 angioplasty because #11i rigidly adhered to V2. (c) After angioplasty.

During surgery, a non-anatomical wedge resection of the lesion was first performed for rapid pathological diagnosis. The patient was diagnosed with adenocarcinoma and was scheduled for right lower lobectomy and systematic nodal dissection. We separated the right middle and lower lobes and identified the common basal artery, V2, and station 11i lymph node, which had an abnormal uptake on PET. Station 11i lymph node was bulky and rigidly adhered to the intermedius bronchus, V2, peripheral A4 + 5, and common basal pulmonary artery (PA).We could not peel off PA and the bronchus without severe damage. Considering respiratory function, we changed the surgery to right middle and lower lobectomy for radical cure and safety operation. Using 3D-MDCT, we identified that A4 + 5 ran between V2 and V1 + 3 (Fig. 2). A4 + 5 was dissected using a stapler. Station 11i lymph node rigidly adhered to V2. To prevent pulmonary congestion due to dissection of V2, angioplasty was performed. However, it was too difficult to perform angioplasty via VATS; therefore, we performed thoracotomy. A partial resection of the V2 wall was conducted using the transverse continuous technique with 6-0 monofilament suture (Prolene). The common basal PA and intermedius bronchus were divided using different staplers (Powered ECHELON gold and Powered ECHELON green, respectively). The postoperative course was uneventful; pathological findings showed minimally invasive adenocarcinoma, and other lymph nodes were negative for malignancy. The pathologic stage was pT1miN0M0 stage I A1. The patient is currently in a good condition and has remained disease free for 2 years.

## 3 Discussion and conclusion

In recent years, VATS lobectomy has become the standard method for lung cancer surgery. In complete VATS, identification of rare anatomical variations is challenging, particularly if prior information is unavailable because the visualization of the surgical field is limited with the use of a thoracoscope. To prevent perioperative complications, detailed surgical simulation must be performed and information should be shared across the team before surgery. Currently, advances in MDCT allow surgeons to easily obtain 3D images of lung structures without expert knowledge [3]. Reportedly, 97.7% of the pulmonary vessels are accurately identified on 3D-MDCT [4], and the use of preoperative 3D-MDCT is more likely to cause a lower incidence of complications [5].

In our case, A4 + 5 ran between V2 and V1 + 3. To the best of our knowledge, this type of anomaly has not been reported yet. 3D-MDCT helps confirm anatomy and any anomalies. Therefore, we could identify A4 + 5 and perform angioplasty without the risk of unexpected bleeding when surgery was changed to middle and lower lobectomy.

Owing to the use of 3D-MDCT, surgery could be safely performed with fewer complications, indicating its usefulness for preoperative assessment owing to its rapidness. This information can be shared across the surgical team simulating extreme familiarity with this rare anatomy.

## Declaration of Competing Interest

None.

## Sources of funding

This research did not receive any specific grant from funding agencies in the public, commercial, or not-for-profit sectors.

## Ethical approval

This is case report is exempt for ethical approval in our institute.

## Consent

Written informed consent was obtained from the patient for publication of this case report and companying images. A copy of the written consent is available for review by the Editorial-in Chief of this journal on request.

## Author contribution

Shinichi Sakamoto: data collection, write the paper.

Hiromasa Matsumoto, Hiroyuki Hino: data interpretation, review and correct the manuscript.

Shoji Sakiyama: review and correct the manuscript.

## Registration of research studies

None.

## Guarantor

Shinichi Sakamoto.

## Provenance and peer review

Not commissioned, externally peer-reviewed.
